# Case of Resistance to Thyroid Hormones With a Relatively Rare Mutation in Thyroid Hormones Receptor

**DOI:** 10.7759/cureus.14911

**Published:** 2021-05-08

**Authors:** Mohanad Ahmed, Khalid Hassan, Hind Ahmed

**Affiliations:** 1 Internal Medicine, Hamad Medical Corporation, Doha, QAT; 2 Department of Endocrinology, Hamad Medical Corporation, Doha, QAT

**Keywords:** thyroid hormones resistance, hypothyroidism, thyroid disorders, thyroid hormone receptor mutation, thrb gene mutation

## Abstract

Normal thyroid hormone level is essential to maintain the normal physiologic function of the human body. Disturbances of these hormone levels have variable clinical manifestations ranging from asymptomatic to severe illness. Resistance to thyroid hormone (RTH) is a syndrome characterized by reduced intracellular action of T3, the active thyroid hormone. It is a rare autosomal dominant condition and occurs mostly due to heterogeneous mutations in the thyroid hormone receptor. Other causes of RTH include thyroid hormone cell membrane transport defect and thyroid hormone metabolism defect. Affected individuals present with symptoms of both increased and decreased thyroid hormone action, depending on the tissue’s predominant receptor isoform expression, the magnitude of hormonal resistance, and the effectiveness of compensatory mechanisms.

Here, we share our experience in diagnosing a case of RTH confirmed with a genetic test and found to have sequence variant mutation that is not well described in the literature previously due to the absence of genetic conclusive evidence.

## Introduction

Thyroid hormones are produced in the response of the thyroid gland to thyroid-stimulating hormone (TSH) secreted from the anterior pituitary gland. Circulating thyroid hormones in the forms of T4 and T3 enter cells by diffusion, and in some tissues, such as the thyroid and brain, by active transport [1]. T3 is the active form of thyroid hormones which will also be available to cells from the local conversion of T4 into T3 inside cells themself. This locally produced T3 can leave the cell and binds to T3 receptors in other tissues. In humans, approximately 80% of extrathyroidal T3 produced from T4 is produced intracellularly [2,3]. Intracellular T3 binds to a nuclear receptor called the thyroid receptor (TR). T3-TR complexes then bind to regulatory regions contained in the genes that are responsive to thyroid hormone and exert action [4].

There are two thyroid hormone receptors (THR), alpha (THRa) and beta (THRb) [4,5]. THRa is mainly found in bones, the intestine, the nervous system, and the heart. While THRb is found mainly in the retina, ear, heart, and nervous system. THRb is the main regulator of the negative feedback on the pituitary thyroid axis [6].

Most patients diagnosed with resistance to thyroid hormone (RTH) are found to have mutations in THRb with multiple variant mutations. However, recently some patients are found to have mutations in THRa [6]. Clinical manifestations depend on the receptor affected and the magnitude of the resistance. There is no specific treatment for RTH. However, after reviewing the literature, we found that a variety of treatment modalities have been tried.

In this article, we are reporting a case of RTH, confirmed with genetic testing, and found to have sequence variant mutation that is not well described due to the absence of genetic conclusive evidence.

## Case presentation

We present a case of a 35-year-old female with a past medical history of type 2 diabetes mellitus on metformin 1 g daily. In 2014, the patient presented with fatigue and weight gain. Clinical examination was completely normal. Basic labs showed normal complete blood count, urine analysis, liver function, and renal function test. Further workup showed a TSH of 23 milli-international units/liter (miu/l) (0.5-5.5 miu/l) and T4 of 15 miu/l (9-21 miu/l). The patient was diagnosed with subclinical hypothyroidism and started on levothyroxine replacement. The starting dose was 25 µg daily and the patient was followed to monitor her symptoms and TSH level.

In the follow-up appointments, the patient reported some improvement in her symptoms. However, her TSH level was persistently elevated. Thyroxin dose was gradually built up till a dose of 100 µg was reached over four years period, but the TSH remained elevated. Other laboratory findings including complete blood count, renal function, liver function, and lipid profile were within normal limits.

During a follow-up appointment in November 2018, TSH was 20 miu/L. Levothyroxine dose was subsequently increased to 150 µg daily. A few weeks later, the patient reported hyperthyroid symptoms including insomnia, tremors, and palpitation. Repeated thyroid function test showed TSH of 6.6 miu/L and T4 level was significantly high (30 mic/L). The dose was reduced to 100 µg and antibodies were requested.

**Figure 1:**
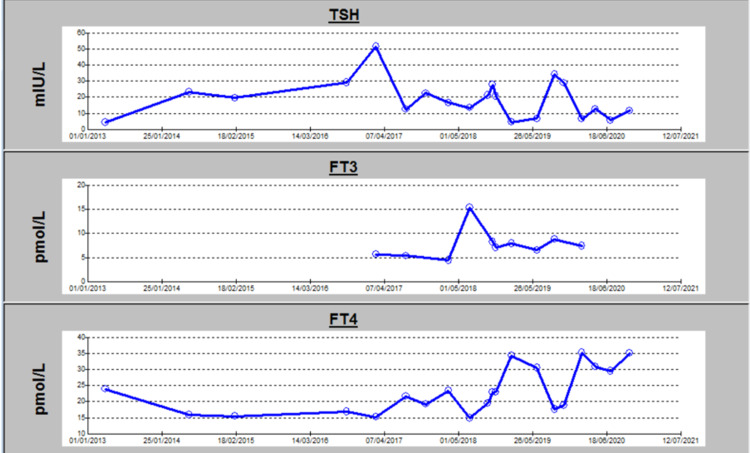
TSH, T4, and T3 levels from diagnosis throughout various thyroxin treatment doses from the time of diagnosis (2013) till 2020.

The next appointment following the reduction of the levothyroxine dose, her insomnia and palpitations improved. Repeated lab tests showed a TSH of 34 miu/L, T4 17 miu/L, antithyroid peroxidase antibody >600 IU/ml (0-34 IU/ml), antithyroglobulin antibody 489 IU/ml (0-115 IU/ml), and TSH receptor antibody 0.8 IU/L (0-1.7 IU/L). Figure 1 showed TSH, T4, and T3 levels throughout treatment with various doses of levothyroxine from the time of diagnosis (2013) till 2020.

Given the values of high TSH not suppressed by levothyroxine treatment, TSH resistance and central causes had to be excluded. A magnetic resonance image (MRI) of the pituitary gland was ordered to exclude TSH secreting pituitary tumor which revealed normal pituitary gland size. The alpha subunit of the pituitary gland which is used as a tumor marker for anterior pituitary gland tumors was also ordered, and it was found to be 0.2 ng/ml (normal < 1.8 ng/ml). Then, a genetic test was ordered to establish a diagnosis of TSH resistance which confirmed TSH resistance through the THRB gene (Table 1).

**Table 1:** A molecular genetic report confirming TSH resistance via THRb gene. THRb: thyroid hormone receptor beta, TSH: thyroid-stimulating hormone. Mode of inheritance: AD: autosomal dominant, AR: autosomal recessive, XL: X linked. CalinVar ID: variant accession. GnomAD: Allele frequency registered in large population database value listed in the highest allele frequency reported within one of seven population categories recognized in GnomAD v.2.0 (the “other” population is excluded). Missense prediction: Summarized output (damaging, conflicting, or tolerated) via polyphen-2, SIFT, mutation taster, and FATHMM [7].

Gene transcript	THRB, NM_001252631.4
Mode of Inheritance, Gene OMIM	AD, AR 190160
DNA variations, predicted effects, zygosity	c.1147C>T, p.Arg383Cys, heterozygous
ClinVar ID	619915
Highest allele frequency in a gnomAD population	Not present
In silico missense prediction	Damaging
Interpretation	Uncertain

The patient was informed of the diagnosis, and the levothyroxine dosage was progressively lowered to the lowest dose necessary to avoid symptoms, with subsequent follow-up based on T4 levels and patient symptoms rather than TSH levels.

## Discussion

Resistance to thyroid hormone was first described as a clinical entity in 1967 [8]. Subsequent studies about the molecular pathogenesis of this syndrome identified mutations in the region of the gene that encodes the ligand-binding domain of THRb. Interestingly, patients with mutations in THRa were not identified until 2012 [6]. Our patient in this article was found to have a mutation in THRb same as the majority of the patients described in the literature.

Patients with RTHb may have some symptoms or signs of hypothyroidism or hyperthyroidism, but these are variable and, when present, often inconsistent. They frequently have elevated thyroid hormone levels, high or normal TSH, and goiter which suggests the importance of THRb in the feedback of the hypothalamic-pituitary axis [6]. In contrast, patients with RTHa present with musculoskeletal and gastrointestinal abnormalities. They usually have near-normal thyroid function due to a lack of THRa contribution in feedback on the hypothalamic-pituitary axis [9,10]. Our patient initially presented with symptoms of hypothyroidism including weight gain and generalized fatigability in addition to high TSH which is consistent with the typical presentation found in the literature. However, she had normal T3 and T4 which is not usually typical in the presentation of RTHb. That is why initially she was labeled as a case of subclinical hypothyroidism.

To date, more than 100 THRB mutations have been reported among RTH patients. Most mutations substitute a single amino-acid residue in the ligand-binding domain [11]. The most unique part of our case is found in the genetic study, which confirmed that the patient is heterozygous in THRb gene for sequence variant designated c.1147C>T, which is predicted to result in amino acid substitution p.Arg383Cys. this variant appears to have been reported in a case report of a french patient with thyroid hormone resistance [12]. It was also reported in another affected individual but was inherited from an apparently unaffected father [11]. Another variant affecting the same amino acid has also been reported in a patient with thyroid hormone resistance [13].

While we suspect this variant could be pathogenic, at this time the clinical significance of this variant is uncertain due to the lack of conclusive functional and genetic evidence.

According to the literature, RTH-beta is associated with an increased risk for autoimmune thyroid disease [14]. That is why patients with RTH-beta should be checked for the presence of thyroid peroxidase and thyroglobulin antibodies. Both antibodies were found to be positive in our patient, so now we are closely monitoring her for the development of other autoimmune diseases.

## Conclusions

Our finding in this case regarding the rare variant mutation in the THRb gene represents an addition to the few data reported about this type of rare mutation before. While we suspect this variant could be pathogenic, at the same time the clinical significance of this variant is uncertain due to lack of conclusive functional and genetic evidence. However, more studies are needed to establish the pathogenicity of this variant.
